# Effect of Pharmaceutical Sludge Pre-Treatment with Fenton/Fenton-like Reagents on Toxicity and Anaerobic Digestion Efficiency

**DOI:** 10.3390/ijerph20010271

**Published:** 2022-12-24

**Authors:** Joanna Kazimierowicz, Marcin Dębowski, Marcin Zieliński

**Affiliations:** 1 Department of Water Supply and Sewage Systems, Faculty of Civil Engineering and Environmental Sciences, Bialystok University of Technology, 15-351 Bialystok, Poland; 2 Department of Environmental Engineering, Faculty of Geoengineering, University of Warmia and Mazury in Olsztyn, 10-720 Olsztyn, Poland

**Keywords:** pharmaceutical sewage sludge (PSS), anaerobic digestion (AD), pre-treatment, biogas, biomethane, advanced oxidation processes (AOPs), Fenton reaction, toxicity

## Abstract

Sewage sludge is successfully used in anaerobic digestion (AD). Although AD is a well-known, universal and widely recognized technology, there are factors that limit its widespread use, such as the presence of substances that are resistant to biodegradation, inhibit the fermentation process or are toxic to anaerobic microorganisms. Sewage sludge generated by the pharmaceutical sector is one such substance. Pharmaceutical sewage sludge (PSS) is characterized by high concentrations of biocides, including antibiotics and other compounds that have a negative effect on the anaerobic environment. The aim of the present research was to determine the feasibility of applying Advanced Oxidation Processes (AOP) harnessing Fenton’s (Fe^2+^/H_2_O_2_) and Fenton-like (Fe^3+^/H_2_O_2_) reaction to PSS pre-treatment prior to AD. The method was analyzed in terms of its impact on limiting PSS toxicity and improving methane fermentation. The use of AOP led to a significant reduction of PSS toxicity from 53.3 ± 5.1% to 35.7 ± 3.2%, which had a direct impact on the taxonomic structure of anaerobic bacteria, and thus influenced biogas production efficiency and methane content. Correlations were found between PSS toxicity and the presence of Archaea and biogas yields in the Fe^2+^/H_2_O_2_ group. CH_4_ production ranged from 363.2 ± 11.9 cm^3^ CH_4_/g VS in the control PSS to approximately 450 cm^3^/g VS. This was 445.7 ± 21.6 cm^3^ CH_4_/g VS (1.5 g Fe^2+^/dm^3^ and 6.0 g H_2_O_2_/dm^3^) and 453.6 ± 22.4 cm^3^ CH_4_/g VS (2.0 g Fe^2+^/dm^3^ and 8.0 g H_2_O_2_/dm^3^). The differences between these variants were not statistically significant. Therefore, due to the economical use of chemical reagents, the optimal tested dose was 1.5 g Fe^2+^/6.0 g H_2_O_2_. The use of a Fenton-like reagent (Fe^3+^/H_2_O_2_) resulted in lower AD efficiency (max. 393.7 ± 12.1 cm^3^ CH_4_/g VS), and no strong linear relationships between the analyzed variables were found. It is, therefore, a more difficult method to estimate the final effects. Research has proven that AOP can be used to improve the efficiency of AD of PSS.

## 1. Introduction

Sludge is widely considered to be one of the organic feedstocks processable using anaerobic digestion (AD) [1]. Both research works and full-scale installations have shown that well-maintained AD can be applied to produce CH_4_ with high efficiency and partially stabilize sludge via removal of putrescible organics, deodorization, improvement of fertilizing properties, and partial sanitization [2,3]. Although AD is well-known, universal and widely recognized as effective, its widespread deployment has been hamstrung by multiple factors [4,5]. One such barrier is the presence of substances that are resistant to biodegradation, inhibit the fermentation process, or are toxic to anaerobic microorganisms [6,7]. Sewage sludge generated by the pharmaceutical sector and other industries is one such substance. Pharmaceutical sewage sludge (PSS) is characterized by high concentrations of biocides, including antibiotics and other compounds that have a negative effect on the anaerobic environment [8] and thus on fermentative bacteria community, impairment or total cessation of metabolism [9], qualitative and quantitative changes in the anaerobic bacterial community [10], and a significant reduction in enzyme activity [11]. This, in turn, results in lower biogas and CH_4_ production, decreased biodegradation, and poorer mineralization of organics [12].

The presence of drugs, antibiotics, and hormones has also become increasingly problematic for municipal sewage sludge processing [13]. This is due to the widespread use of medicinals in various industries (crop farming, livestock farming, aquaculture, feed production, etc.) and the use of biocides/antibiotics in households [14,15]. It is estimated that there are approx. 200,000 pharmaceuticals (defined as substances intended to have a therapeutic, preventive, or diagnostic effect) in the world today, though the figure is much smaller for singular countries, ranging from 5000 to 10,000 individual substances [16]. Other sources state that the number of chemically active compounds used in pharmaceuticals is 4000 in Europe alone [17]. The pharmaceutical industry is one of the fastest growing industries, with total global revenues breaking through the 1 trillion USD barrier for the first time in 2014. From 2017 onwards, the market has been growing at 5.8% per annum, with total global revenues reaching 1143 billion USD in 2017 and 1462 billion USD in 2021. The bulk of this income was generated in North America, owing to the dominant position of the American pharmaceutical industry. However, it was the Chinese pharmaceutical industry that recorded the fastest growth among the national markets in recent years [18].

Since the problem of pharmaceuticals in sewage sludge only started to draw attention relatively recently, the popular sludge treatment technologies are not equipped to remove and reduce their negative effects [19]. There are also no legal regulations that could force utilities to implement such solutions [20]. Thus, there is a pressing need to alleviate and remove those properties of PSS that hamper its use as AD feedstock by improving current technologies and exploring new ones. Such technologies should serve to alleviate toxicity, remove digestion inhibitors and improve anaerobic digestibility (thus resulting in better methane yields and stabilization performance). The key criteria for selecting a process should not be limited to the pollutant removal rate and lack of unwanted by-products—economic aspects should be considered as well.

There are processes successfully used in water and sewage treatment that could serve as an alternative and complement to existing methods, such as those based on intensive oxidation [21]. Such advanced oxidation processes (AOPs) include what is known as the Fenton reaction, mediated by H_2_O_2_ with Fe ions as the catalyst [22,23]. In the course of the reaction, H_2_O_2_ is catalytically degraded in the presence of Fe^2+^ or Fe^3+^ ions, producing reactive free hydroxyl radicals (OH^•^) with a very high oxidation potential (2.8 V) [24]. The nature of the Fenton reaction and the research to date hint at this method being a competitive addition to sludge treatment [25]. AOP facilitates the chemical degradation of hard-to-biodegrade pollutants, toxic substances, biocides and antibiotics [26]. The literature data point to the Fenton reaction as useful means of organic removal, deodorization, color removal, and sanitization [27,28]. AOPs tend to be used either in standalone systems for efficient water and sewage treatment or as a part of an integrated process for increased performance [29,30].

The reactivity of (OH^•^) and its high oxidation potential make AOP a suitable method for breaking down biodegradation-resistant substances, including those contained in sewage from the production of chemicals, pharmaceuticals, [31], insecticides [32], colorants [33], explosives [34], leather processing [35], refinery/filling-station waste [36], specialty chemicals (such as plastics production and adhesive products), and timber impregnation [37,38]. Fenton products can oxidize most carbon species, including very complex and hard-to-degrade ones. Oxidizable compounds include phenols, ketones, alcohols, benzene, nitrobenzene, perchloroethylene, toluene, *p*-toluene, aniline, *p*-nitrophenol, humic substances, and formaldehyde [39].

The aim of this study was to assess the applicability of AOPs via Fenton/Fenton-like reactions in the pre-treatment of pharmaceutical sewage sludge (PSS) intended for anaerobic digestion. It tested the extent to which the method reduced PSS toxicity and improved AD performance (biogas yields and methane fractions). The results were used to develop optimization methods to estimate AOP methane production as a function of chemical reagent doses and sludge parameters.

## 2. Materials and Methods

### 2.1. Experimental Design

**Figure 1:**
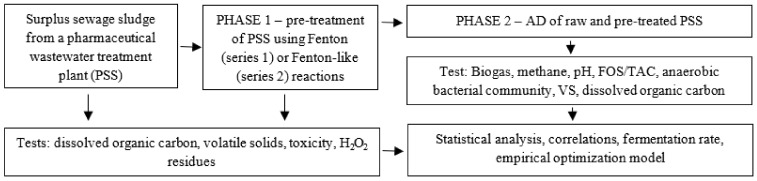
Flowchart of the experiment, tests and calculations.

The Surplus sewage sludge from a pharmaceutical sewage treatment plant (PSS) was used for the study. The experiment was divided into two phases. In phase 1 (P1), the PSS was pre-treated through the advanced oxidation process (AOP). During phase 2 (P2), the PSS was subjected to anaerobic digestion (AD). Phase 1 was executed in two series with different levels of AOP chemical reagents. The Fenton reagent (Fe^2+^/H_2_O_2_) was tested in series 1 (S1), and a Fenton-like regent (Fe^3+^/H_2_O_2_) in series 2 (S2). A flowchart of the process is given in Figure 1.

Each series was divided into six variants (V) with different doses of chemical reagents applied to the PSS. The experimental design is presented in Table 1.

### 2.2. Materials

#### 2.2.1. Sourcing of Fe and H_2_O_2_ Ions

Ferrous Fe^2+^ ions were used as FeCl_2_·4H_2_O (Sigma-Aldrich, Taufkirchen, Germany), yellowish-white crystalline powder, purity ≥ 99.5%, sulfates ≤ 0.02%, iron (Fe^3+^) ≤ 0.01%, and arsenic (As) ≤ 2 ppm. Ferric Fe^3+^ ions were used as FeCl_3_·4H_2_O (Sigma-Aldrich, Taufkirchen, Germany), purity ≥ 99.0%, sulfates (SO_4_) ≤ 0.03%, lead (Pb) ≤ 0.01%, copper (Cu) ≤ 0.01%) and H_2_O_2_ as a 30% solution of perhydrol (Merck, Darmstadt, Germany), colorless liquid, faint odor, pH 2–4, density 1.11 g/cm^3^.

#### 2.2.2. Pharmaceutical Wewage Sludge (PSS) and Anaerobic Sludge (AS)

Gravity-thickened PSS was extracted from the secondary clarifier of an aerobic pharmaceutical wastewater treatment plant (P-WWTP) running a conventional activated sludge process without enhanced nutrient removal. Wastewater directed to P-WWTP was from a pharmaceutical company that produces 42 active substances, including baclofen, aripiprazole, tadalafil, vardenafil, alendronate, risedronate, sildenafil, hydrochlorothiazide, xylometazoline, sildenafil, piracetam, pentoxifylline, metronidazole, hydrochlorothiazide, and ethopyrine. The sewage flow rate averaged 2000 m^3^/d. The P-WWTP generates approx. 30 tonnes of PSS/day. The anaerobic sludge (AS) inoculum was sourced from the enclosed digesters of the Municipal Water Treatment Plant in Olsztyn (Poland). The digester operating parameters are organic load rate (OLR) approx. 2.4 kg VS/m^3^·d, hydraulic retention time (HRT) 20 days, and temperature 35ºC. Prior to the experiment, the AS was conditioned and adapted for PSS anaerobic digestion for 60 days (three times hydraulic residence time in the reactor). The characteristics of the PSS and AS used in the study are presented in Table 2.

### 2.3. Experimental Set-Up

#### 2.3.1. Phase 1—AOP Reactor

**Figure 2:**
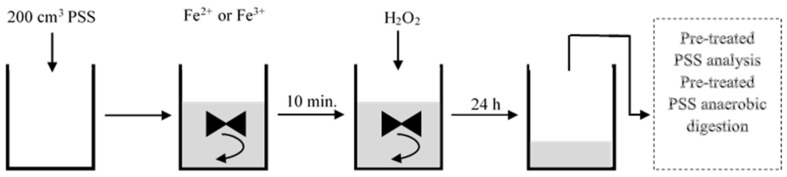
Scheme of the experiment organization in Phase 1—AOP reactor.

At the beginning of the P1 experimental cycle, 200 cm^3^ PSS was fed into the reactor, after which the chemical reagents were introduced. The PSS was first amended with the target Fe dose, then, after 10 minutes, with the H_2_O_2_ at a constant Fe/H_2_O_2_ ratio of 1:4 by weight. The sludge was agitated for 20 min at 150 rpm with vertical, 3-blade, mechanical mixers (Nanostar 7.5 Digital, IKA, Poland) to ensure uniform reagent distribution, then at 50 rpm to allow the PSS to react thoroughly with the reagent. The sludge retention time in the reactor was 24 h. The scheme of the experiment organization in this phase is shown in Figure 2. In phase 2, the PSS was subjected to AD.

#### 2.3.2. Phase 2—Anaerobic Reactors

AD performance was tested using the AMPTS II kit (Bioprocess Control, Lund, Sweden). The semi-batch process was run in reactors with a total volume of 2.0 dm^3^ fitted with vertical, 3-blade mechanical mixers rotating at 100 rpm (3 min ON/10 min OFF regime). Prior to AD, the reactors were inoculated with 1.0 dm^3^ anaerobic sludge. The organic load rate was 2.5 g_VS_/dm^3^ d; the hydraulic retention time was 20 d. Digested sludge was removed once a day, and the reactor was replenished with an equivalent amount of feedstock. In order to ensure anaerobic conditions at the start of the experiment, the inoculum + PSS mixture was purged with pure nitrogen for 3 min. The temperature was kept at a constant 40 °C by placing the reactors in a water bath. Bioreactors were fitted with a nozzle in the carbon dioxide absorption unit. The resultant biogas was fed into a 100 cm^3^ tank filled with a 3M solution of NaOH (Pol-Aura Ltd., Olsztyn, Poland).

### 2.4. Analytical Methods

TS, VS and MS were determined gravimetrically. TS levels in the biomass were determined by drying to a constant weight at 105 °C, then burning it at 550 °C (the loss on ignition was the VS, as per PN-EN 15935: 23022-01) [40]. Biomass samples desiccated at 105 °C were assayed for TC, TOC and TN using a Flash 2000 elemental particle analyzer (Thermo Scientific, USA) [41]. The concentrations of dissolved TOC were quantified using a TOC-L (Shimadzu, Kyoto, Japan) [42]. TP was determined colorimetrically in ammonium metavanadate (V) and ammonium molybdate after sample mineralization in a mixture of sulfuric (VI) and chloric (VII) acids at 390 nm, using a DR 2800 spectrophotometer (HACH Lange, Weilheim, Germany) [43]. Total protein was calculated by multiplying the value of TN by the protein conversion factor of 6.25 [44]. Reducing sugars were determined colorimetrically with anthrone reagent at 600 nm using a DR 2800 spectrophotometer (HACH Lange, Weilheim, Germany) [45]. Lipids were extracted using the Soxhlet method with a Buchi extraction apparatus (Flawil, Switzerland) and then determined by weight difference afterward [46]. The pH value of H_2_O was determined potentiometrically with an 867 pH Module (Metrohm, Herisau, Switzerland) [47]. The FOS/TAC (the ratio of the buffer capacity of the sample to the VFA levels in the sample) was determined using a TitraLab AT1000 titrator (HACH Lange, Weilheim, Germany) [48]. Unreacted H_2_O_2_ was quantified iodometrically and with the use of Quantofix Peroxide strips (Macherey–Nagel, Düren, Germany) (range: 1–100 and 50–1000 mg/dm^3^) [49]. Acute toxicity of the sludge (aqueous extracts) was measured using *Vibrio fischeri* bacteria in an M 500 Analyzer (Azur Environmental, Delaware, USA), acc. to PN-ISO 11348-2:2008 [50]. The aqueous extract was prepared by adding four volumes of distilled water on top of one volume of sludge and agitating mechanically for 24 h [51]. The molecular analysis aimed to determine the percentage of AD bacteria in the biomass using the fluorescent in situ hybridization (FISH) technique [52]. Four molecular probes were used for hybridization: a Bacteria-universal probe EUB338 [53], an Archaea-universal probe ARC915 [54], a *Methanosarcinaceae*-targeting probe MSMX860, and a *Methanosaeta*-targeting probe MX825 [55]. The composition of biogas was measured using a gastight syringe (20 mL injection volume) and a gas chromatograph (GC, 7890A Agilent, Santa Clara, CA, USA) equipped with a thermal conductivity detector (TCD) [56]. The GC was fitted with the two Hayesep Q columns (80/100 mesh), two molecular sieve columns (60/80 mesh), and a Porapak Q column (80/100) operating at a temperature of 70 °C. The temperature of the injection and detector ports were 150 °C and 250 °C, respectively. Helium and argon were used as the carrier gases at a flow of 15 mL/min. Additionally, biogas was analyzed by the GMF 430 Gas Data analyzer. The content of methane (CH_4_) and carbon dioxide (CO_2_) was measured. The validated analytical procedure was calibrated against a standard curve. Therefore, the dependence of the analytical signal (peak area) as a function of the concentration of the analyzed component was determined for a series of standard gas mixtures.

### 2.5. Calculation Methods

The digestion coefficient (portion digested), i.e., the ratio of the organic VS load removed in the reactor to the VS load fed into the reactor, was determined using the following equation:

(1) $$\eta_{F}=\frac{VS_{in}\cdot \rho_{in}\cdot Q_{in}-VS_{out}\cdot \rho_{out}\cdot Q_{out}}{VS_{in}\cdot \rho_{in}\cdot Q_{in}}$$

where $\eta_{F}$—digestion coefficient, %; $VS_{in}$—concentration of organic compounds in the influent, g/dm^3^; $VS_{out}$—concentration of organic compounds in the digestate, g/dm^3^; $\rho_{in}$—influent density, g/cm^3^; $\rho_{out}$—digestate density, g/cm^3^; $Q_{in}$—daily volume of feedstock (in), cm^3^/d; $Q_{out}$–daily volume of digestate (out), cm^3^/d.

Biogas/CH_4_ production per *VS* load was calculated as follows:

(2) $$Y_{b/CH_{4}}^{VS_{rem}}=\frac{V_{b/CH_{4}}}{(VS_{in}\cdot \rho_{in}\cdot Q_{in}-VS_{out}\cdot \rho_{out}\cdot Q_{out})/1000}$$

Biogas/CH_4_ production per *VS* load in the influent was calculated using the following equation:

(3) $$Y_{b/CH_{4}}^{VS_{in}}=\frac{V_{b/CH_{4}}}{(VS_{in}\cdot \rho_{in}\cdot Q_{in})/1000}$$

$Y_{b/CH_{4}}^{VS_{rem}}$—biogas production per *VS_rem_*, cm^3^/g*VS_rem_*; $Y_{b/CH_{4}}^{VS_{in}}$—biogas production per *VS* in the influent, cm^3^/g*VS_in_*; $V_{b/CH_{4}}$—volume of biogas/CH_4_ produced per influent load, cm^3^/d; $Q_{in}$—single load of influent (by volume), cm^3^; $Q_{out}$—specific post-AD digestate out (by volume) cm^3^.

### 2.6. Statistical Methods and Optimization

All experimental variants were conducted in triplicate. Statistical analysis of the results was carried out using STATISTICA 13.1 PL package. The Shapiro–Wilk test was used to verify the hypothesis regarding the distribution of every researched variable. ANOVA was performed to establish the significance of differences between variables. The homogeneity of variance was determined using Levene’s test. Significant differences between variants were determined via Tukey’s honestly significant difference (HSD) test. A significance level of α = 0.05 was adopted for the tests.

Empirical equations were elaborated using stepwise regression with multiple regression. The equations were then used to estimate the correlation between the amount of methane and the post-AOP PSS parameters. Predictors having a significant impact on the changes in estimated parameters were determined in model systems. In addition, the accuracy of the models’ fit to empirical data was estimated via a coefficient of determination. The significance of multiple regression models was verified by the F-test. A lack-of-fit test was conducted to evaluate whether the proposed models are sufficiently detailed by comparing the proposed models with full models (which included the remaining explanatory variables omitted in the proposed models). The developed models were subjected to estimation. Next, their fit to obtained results was evaluated by analysis of residuals. The assumption of normality of residual distribution was verified, and the models’ accuracy was evaluated by deleting the residual values with respect to predicted values (Statistica 13.1 PL).

## 3. Results and Discussion

### 3.1. Phase 1—Pre-Treatment Efficiency

#### 3.1.1. Organic Compounds

The adopted method of pre-treatment had no significant effect on volatile solid (VS) levels in the PSS, with VS values being similar across all series and variants (*p* > 0.05). VS in raw PSS was 78.7 ± 1.9 % TS (Table 3). In S1 (Fe^2+^/H_2_O_2_), the VS levels ranged from 77.1 ± 2.7% TS (V1) to 74.4 ± 2.0% TS (V4). In S2 (Fe^2+^/H_2_O_2_), the range was between 78.5 ± 0.4% TS (V1) and 76.3 ± 0.5% TS (V5). Trends in VS in the PSS are presented in Table 3. Atay and Akbal [57] have demonstrated that AOP can be used for efficient stabilization of sludge from municipal wastewater treatment plants, removing 26.8% VS using 0.07/1 Fe^2+^/H_2_O_2_ and 60 g/kgTS H_2_O_2_ [57]. Other researchers have reported successful experiments on textile sludge [58] and anaerobically digested sludge [59], among others. The failure to remove VS from the PSS most likely stems from insufficient oxidizing power of the reagents at the doses used [60]. Researchers have suggested that, in cases such as this, free hydroxyl radicals initially react with dissolved substances [61]. This is corroborated by Fontmorin and Sillanpää [59]. However, it is important to note that VS removal from sludge through pre-treatment could negatively affect the biomethane yield if the sludge is to be used as feedstock for AD [62]. 

Dissolved TOC was 326 ± 10 mg/dm^3^ in the control group, 296 ± 5 mg/dm^3^ (the lowest) in S1V5, and 303 ± 8 mg/dm^3^ in S2V5. The trends in dissolved TOC in the tested PSS are presented in Table 3. The TOC values were significantly different from the raw PSS (*p* < 0.05). Multiple studies have shown the Fenton and Fenton-like reactions to be highly effective at removing dissolved organics [63,64]. This has been demonstrated in treatments of various sewage and leachates [65,66,67]. There have also been reports of AOP inducing the partial breakdown of complex organics, which did not affect the TOC results but did result in better biodegradation of pollutants and lower toxicity [68]. One example of this phenomenon in practice has been presented by Catalkaya and Kargi [69], who tested the advanced oxidation of diuron in an aqueous solution by Fenton’s reagent. The authors found that only 58% of diuron was mineralized after 240 min under optimal operating conditions, indicating the formation of certain intermediate products. No effect of H_2_O_2_ and Fe(II) on TOC removal was found [69]. Similar trends have been reported by Pérez et al. [70] in processing waste from paper pulp treatment effluents.

#### 3.1.2. Toxicity

**Figure 3:**
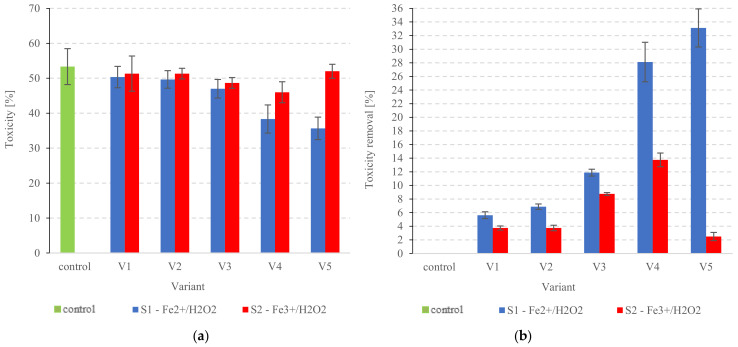
Toxicity after pre-treatment (**a**) changes in PSS, (**b**) removal.

This study shows that Fenton’s reagent (Fe^2+^/H_2_O_2_) significantly reduced the toxicity of the tested PSS. The toxicity of raw PSS was 53.3 ± 5.1%, but significant changes (*p* < 0.05) were noted very early into the pre-treatment, with toxicity dropping to S1V2 at 49.6 ± 2.5% (Figure 3a). The lowest statistically comparable levels (*p* > 0.05) were found in S1V4 and S1V5 at 38.3 ± 4.0% and 35.7 ± 3.2%, respectively (Figure 3a). Accordingly, these were also the series with the highest toxicity removal rates (28.1 ± 2.9% and 33.1 ± 2.8% removal, respectively) (Figure 3b). The use of Fe^2+^/H_2_O_2_ reagents for reducing the toxicity of biodegraded feedstock has also been successfully tested in other studies [71]. Barbusiński and Filipek [72] demonstrated that Fenton’s reagent could completely eliminate the high toxicity of industrial wastewater. Similarly, Gerulová et al. [73] found that preliminary treatment with Fe^2+^:H_2_O_2_ at a 1:10 molar ratio can reduce the toxicity of metalworking wastewater fluids (MWF), improving their biodegradability [73]. Finally, Lin et al. [74] have obtained reduced toxicity in acrylic fiber manufacturing wastewater. The reduced toxicity brought on by the Fenton treatment is explained by its ability to convert refractory organic matter to smaller organic/inorganic molecules [75].

PSS treatment with Fe^3+^/H_2_O_2_ showed highly variable performance in the present study. Significant toxicity reduction (*p* < 0.05) was achieved only in S2V3 and S2V4 (48.7 ± 1.5% and 46.0 ± 3.0%, respectively) (Figure 3a). The highest tested dose of the reagents (S2V5) resulted in higher toxicity levels at 52 ± 2% (*p* > 0.05) (Figure 3a). This may be attributable to the two-step nature of OH^•^ production in the Fenton-like process [76]. The first step generates OH_2_^•^ and reduces Fe^3+^ ions to Fe^2+^ [77]. It is only in the second stage that the typical Fenton reaction takes over, with a catalytic decomposition of H_2_O_2_ to free hydroxyl radicals [78]. Therefore, a Fenton-like reaction may be slower and less effective at removing organics and reducing toxicity [79,80]. Fe^2+^/H_2_O_2_ has been shown to perform better than Fe^3+^/H_2_O_2_ at treating textile dyeing wastewater [81]. The more complex nature of Fenton-like reactions poses a risk of incomplete degradation of H_2_O_2_ to OH^•^ and the presence of residual H_2_O_2_ in the medium [82]. H_2_O_2_ has a high oxidation potential, making it harmful to microorganisms [83]. Arslan-Alaton and Gurses tested the Fenton-like oxidation of antibiotic formulation effluent and found high levels of residual H_2_O_2_, which they attributed to the relatively slow and poor COD reduction kinetics [84].

#### 3.1.3. Residual (Unreacted) H_2_O_2_ and pH Changes in the PSS

**Figure 4:**
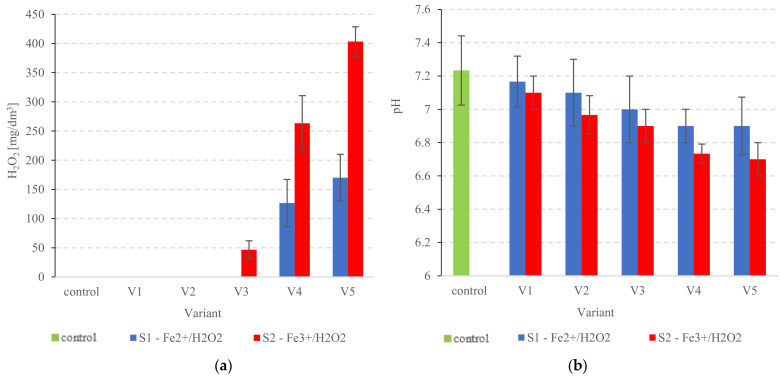
In the PSS after pre-treatment, (**a**) residual H_2_O_2_ levels and (**b**) changes in pH.

No residual H_2_O_2_ was detected in the Fe^2+^/H_2_O_2_ variants S1V1–S1V3 (Figure 4a). Some amounts were found in S1V4 (126.7 ± 40 mgH_2_O_2_/dm^3^) and S1V5 (170 ± 40 mgH_2_O_2_/dm^3^) (Figure 4a). In contrast, the Fenton-like reaction produced higher rates of residual H_2_O_2_. Traces of unreacted oxidant were found in the PSS quite early into the experiment (S2V3) at 46.7 ± 15.3 mgH_2_O_2_/dm^3^, rising concurrently with the reagent doses in the subsequent variants (Figure 4a). Increases to 263.3 ± 47.2 mgH_2_O_2_/dm^3^ and 403.3 ± 25.2 mgH_2_O_2_/dm^3^ were found in S2V4 and S2V5, respectively (Figure 4a). The presence of residual H_2_O_2_ in media processed using AOP (via Fenton or Fenton-like reactions) has been found by other studies as well [85,86,87]. Verma and Haritash [85] used this process to remove amoxicillin (AMX) wastewater and found that treatment with 375 mg/dm^3^ H_2_O_2_ left 34 mg/dm^3^ residual H_2_O_2_. Further increases of H_2_O_2_ levels (450 and 600 mg/dm^3^) reduced the degradation rate [85]. The presence of residual H_2_O_2_ may be an indication of reduced oxidation capacity in the Fenton system and a diminished degradation rate, possibly brought on by the removal of free hydroxyl radicals and generation of the HO^•^_2_ radical [86,87]. 

The use of salt as an Fe ion donor for radical formation can also lower the pH of the treated media. This has been shown to play a particularly large role when using iron-sulfur compounds such as Fe_2_(SO_4_)_3_, FeClSO_4_, and FeSO_4_·7H_2_O [88,89]. The Fenton reaction is more efficient at low pH, as demonstrated by Alalm et al. [90] and Verma and Haritash [85]. The authors posited that the best performance was achieved at the range of pH from 2 to 4, with pH 3 being the most optimal choice [85]. However, it is important to note that lowering pH of sewage sludge intended to be anaerobically digested can be detrimental to digestion performance [91]. Methanogenic bacteria are sensitive to changes in the environment and require neutral pH for optimal metabolic activity [92]. With this in mind, we used chlorides as Fe ion donors in our study, as they do not produce significant reductions in pH in pre-treated PSS. In the Fenton reaction group, the pH varied across experimental variants—from 7.16 ± 0.15 in S1V1 to 6.90 ± 0.17 in S1V5 (*p* > 0.05) (Figure 4b). The Fe^3+^/H_2_O_2_ series also showed some variance—from 7.10 ± 0.10 (S2V1) to 6.70 ± 0.10 (S2V5) (*p* > 0.05) (Figure 4b). By comparison, the raw PSS had a pH of 7.23 ± 0.21 (Figure 4b). There have been reports of highly advanced oxidization performance in near-neutral media. This includes a study by Chen et al. [93], where efficient degradation of wastewater tetracycline was achieved via a Fenton-like reaction at pH between 4 and 8 [93]. Another study [94] showed 100% removal of Rhodamine B (RhB) and 90% removal of tetracycline (TC) by a Fenton-like reaction at neutral pH [94].

### 3.2. Phase 2—Anaerobic Digestion Performance

#### 3.2.1. Biogas and Methane Production

**Figure 5:**
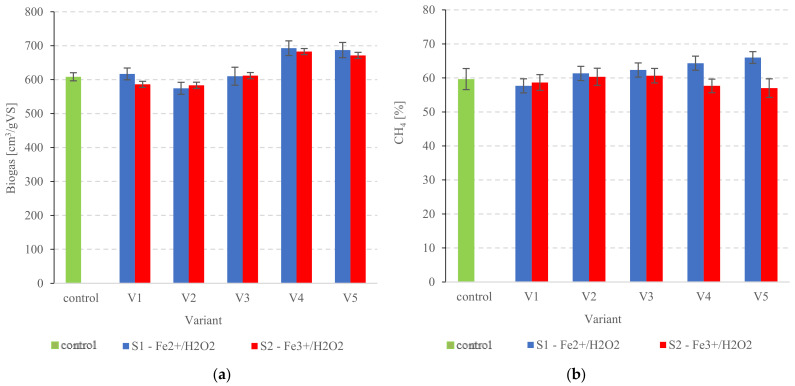
(**a**) Biogas production from pre-treated PSS, (**b**) CH_4_ concentration in the biogas.

The total biogas yield from the non-treated PSS was 608.5 ± 11.9 cm^3^/gVS (Figure 5a). Productivity was boosted by the highest chemical reagent doses in the PSS across all AOP variants. Variants S1V4 and S1V5 in the Fe^2+^/H_2_O_2_ group produced 692.8 ± 21.6 cm^3^/gVS and 687.3 ± 22.4 cm^3^/gVS, respectively (Figure 5a). The Fe^3+^/H_2_O_2_ system yielded 682.7 ± 12.1 cm^3^/gVS (S2V4) and 671.4 ± 9.1 cm^3^/gVS (S2V5) biogas (Figure 5a). The CH_4_ production was higher both in terms of fraction in the biogas and nominal production rate. The digestion coefficient $\eta_{F}$ for the control was 43.15 ± 2.2% (Table 4). The highest coefficients were obtained for variant 4, both in the Fenton group and the Fenton-like group—59.18 ± 2.3% in S1V4 and 49.04 ± 3.4% in S2V4 (Table 4). The CH_4_ fraction produced in the PSS control group was around 59.7 ± 3.1% (Figure 5b). However, the CH_4_ fractions in the biogas were significantly higher (*p* < 0.05) in the Fenton group, with the highest statistically comparable values noted in S1V4 and S1V5 at 64.3 ± 2.3% and 66.0 ± 1.7%, respectively (Figure 5b). Others showed no statistically significant differences from the control (*p* > 0.05), with CH_4_ concentrations of 57.7 ± 2.1% (S1V1) to 62.3 ± 1.9% (S1V3) (Figure 5b). In contrast, the Fenton-like reagent did not produce significant changes (*p* > 0.05) in CH_4_ levels in the biogas—the fractions were similar across all variants and ranged from 57.0 ± 2.6% (S2V5) to 60.7 ± 2.5% (S2V3) (Figure 5b).

Zawieja and Brzeska also managed to improve biogas yields from the AD of surplus sludge by advanced oxidation of sludge with Fenton’s reagent [95], finding that the optimal process parameters were: Fe ion dose = 0.08 g Fe^2+^/gTS and Fe^2+^:H_2_O_2_ ratio = 1:5. This configuration produced a biogas yield of 0.53 dm^3^/gVS (which represents a 35% increase in biogas production against the unprocessed sludge) and a digestion coefficient of 59%. The methane fraction in the biogas was unaffected, however, remaining at 70% [95]. A massive increase in biogas production—75% against the control—was achieved by Dewil et al. [96] by pre-treating surplus sludge with Fenton’s reagent at a dose of 0.07 Fe^2+^/g H_2_O_2_, 50 mg H_2_O_2_/kgTS. Methane content in the biogas ranged between 65 and 70%, with its calorific value remaining unaltered [96].

**Figure 6:**
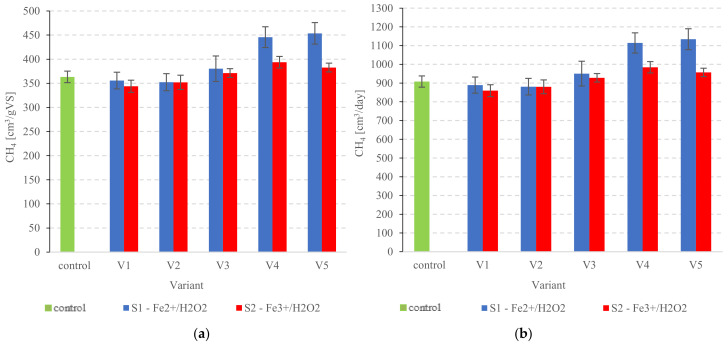
CH_4_ production (**a**) from pre-treated PSS, (**b**) daily.

The CH_4_ yield from raw PSS was around 363.2 ± 11.9 cm^3^/gVS (Figure 6a). Variants S1V1 and S1V2 failed to perform significantly better (*p* < 0.05) in terms of anaerobic digestion at 355.7 ± 17.3 cm^3^/gVS and 352.3 ± 17.7 cm^3^/mgVS, respectively (Figure 6a). Considerably higher (*p* < 0.05) CH_4_ yields of approx. 450 cm^3^/gVS—the highest in the present study—were obtained in S1V4 and S1V5 (Figure 6a). Conversely, the Fe^3+^/H_2_O_2_ group showed significantly poorer anaerobic digestion performance compared with S1 (*p* < 0.05). The highest CH_4_ yields at 393.7 ± 12.1 cm^3^/gVS were found for S2V4 (Figure 6a). Lower levels of gas metabolites were noted for S2V5, with metabolic bacteria outputting 382.7 ± 9.1 cm^3^/gVS (Figure 6a). CH_4_ production in variants S2V1—S2V3 ranged from 352.0 ± 12.6 cm^3^/gVS to 371.2 ± 9.1 cm^3^/gVS (Figure 6a). These values are statistically comparable to those determined for non-pretreated PSS (*p* > 0.05). S1V4 was the best-performing variant in terms of daily CH_4_ production at 1114.2 ± 54.0 cm^3^/d (Figure 6b). The effect of Fenton’s reagent dose on AD of surplus sludge was also investigated by Dhar et al. [97]. With Fe^2+^:H_2_O_2_ ratios of 1.5/0.6, 1.5/1.5, 3/0.6, 2.5/1.5, 2.5/2, and 3/2, the experiment yielded 258 ± 1 cm^3^/gVS_Sadded_, 254 ± 1 cm^3^/gVS_Sadded_, 253 ± 4 cm^3^/gVS_Sadded_, 260 ± 1 cm^3^/gVS_Sadded_, 260 ± 1 cm^3^/gVS_Sadded_, and 251 ± 6 cm^3^/gVS_Sadded_ methane, respectively, compared with the 226 ± 4 cm^3^/gVS_Sadded_ methane produced by the control. Likewise, Erden and Filibeli [98] found that AD reactors produced more methane when fed with surplus sludge preconditioned with Fenton’s reagent at 0.067 g Fe^2+^/g of H_2_O_2_ and 60 g H_2_O_2_/kgTS—whereas the control yielded 400.3 cm^3^/gVS methane, the Fenton-processed group produced 547.3 cm^3^/gVS. (1.4 times higher methane output) [98]. Other researchers [99,100] have further demonstrated that a Fenton oxidation process and anaerobic digestion can be an effective combination, as the posited pre-treatment methodology significantly increases the rate of AD, thus boosting feedstock biodegradability and methane production.

#### 3.2.2. Bacterial Community Structure

This study also tested the effect of the PSS pre-treatment variant on the taxonomic structure of anaerobic bacteria communities, as presented in Table 5. Bacteria (EUB338) formed the bulk of the microbes in the bioreactor with non-pretreated PSS, accounting for 69 ± 10% of the community. Methanogenic Archaea (ARC915), *Methanosarcinaceae* (MSMX860) and *Methanosaeta* (MX825) accounted for 23 ± 9%, 11 ± 5%, and 6 ± 2%, respectively. The conventional Fenton reaction significantly increased (*p* < 0.05) the share of methanogenic microbes in the bacterial community. Variants S1V1 to S1V3 had 26 ± 6% Archaea, 11 ± 4 − 13 ± 6% *Methanosarcinaceae* (MSMX860) and 7 ± 3 − 8 ± 4% *Methanosaeta* (MX825). In variants S1V4 and S1V5, the proportion of Archaea rose above 30%, *Methanosarcinaceae* (MSMX860) ranged from 13 ± 5 to 14 ± 6%, and *Methanosaeta* (MX825) accounted for 10 ± 4%. Pre-treatment with the Fe^3+^/H_2_O_2_ reagent produced no statistically significant effects on the taxonomic structure of the microbes (*p* > 0.05). The shares of Archaea (ARC915), *Methanosarcinaceae* (MSMX860), and *Methanosaeta* (MX825) fell within the narrow ranges of 19 ± 5 − 23 ± 6%, 9 ± 4 − 12 ± 6%, and 6 ± 2 − 9 ± 4%, respectively. Singh et al. [101] have argued that hydroxyl radicals introduced by Fenton’s reagents can trigger direct DNA alterations, as well as other genotoxic effects on cells, with methanogen activity being particularly susceptible.

#### 3.2.3. Organic Compounds

**Figure 7:**
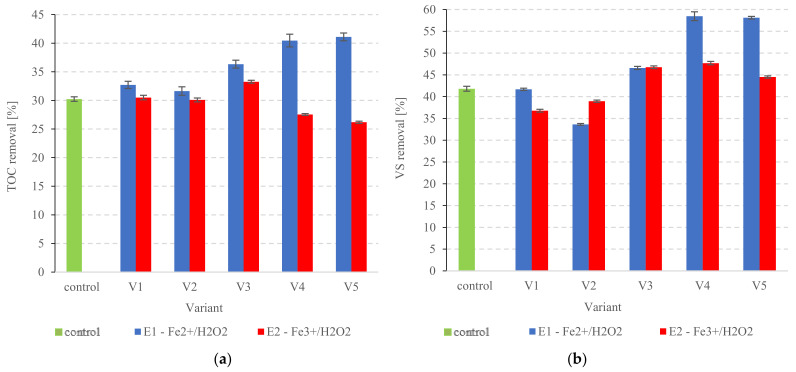
(**a**) TOC removal (**b**) VS removal from the PSS after pre-treatment.

**Figure 8:**
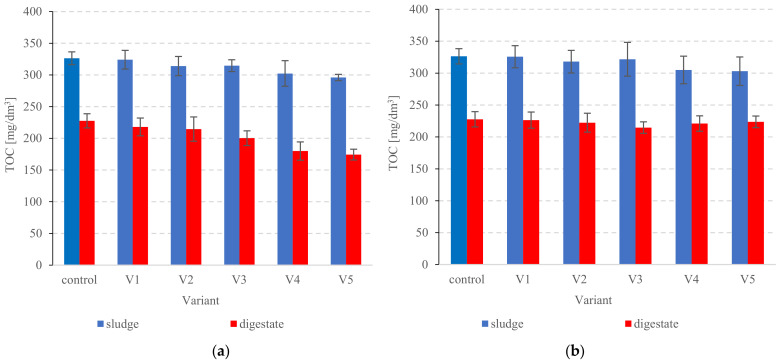
TOC in the sludge and digested PSS in (**a**) series 1, and (**b**) series 2.

**Figure 9:**
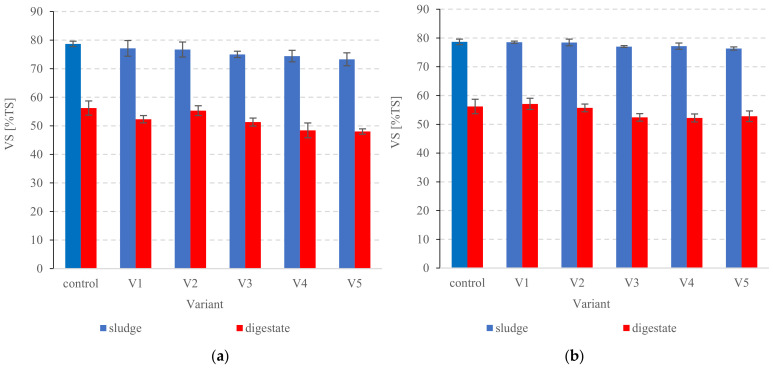
VS in the sludge and digested PSS in (**a**) series 1, (**b**) series 2.

The TOC removal from the dissolved phase was monitored during the anaerobic digestion of PSS (Figure 7a). The Fe^2+^/H_2_O_2_ variants performed significantly better in terms of biodegradation (*p* < 0.05). Furthermore, as Fenton’s reagent doses increased, so did the biodegradation efficiency. Similar correlations were observed for the anaerobic biodegradation of VS (Figure 7b). Dissolved TOC removal for the non-pretreated sludge was 30.2 ± 0.4% (Figure 7a). In variants S1V1 to S1V3, the removal ranged from 31.6 ± 0.7% to 36.3 ± 0.6% (Figure 7a). The highest statistically comparable performance (*p* > 0.05) was achieved in variants S1V4 and S1V5 and reached 40.5 ± 1.1% and 41.1 ± 0.8%, respectively (Figure 7a). Dissolved TOC levels were between 227.7 ± 11.1 mg/dm^3^ in control and 174.3 ± 8.5 mg/dm^3^ in S1V5 (Figure 8a). The Fenton-like group showed significantly poorer dissolved TOC removal—from 26.2 ± 0.2% (S2V5) to 33.3 ± 0.3% (S2V3) (Figure 7a). Nominal levels ranged from 214.7 ± 5.5 mg/dm^3^ to 226.3 ± 5.5 mg/dm^3^ (Figure 8b). For comparison, 41.8 ± 0.6% VS was removed in the control group (Figure 7b). Variant S1V4 (Fenton process) performed the best in terms of VS removal with a removal rate of 58.5 ± 1.0% (Figure 7b). VS levels ranged from 56.2 ± 2.5%TS in control to 48.0 ± 0.9% TS in S1V5 (Figure 9a). The highest performance among the Fenton-like variants was recorded for S2V4 at 47.7 ± 0.4% (Figure 7b). The final (post-AD) VS was between 56.2 ± 2.5% TS in the control and 52.2 ± 1.4%TS in S2V4 (Figure 9b). Other researchers have also noted improved organics removal when combining Fenton pre-treatment with AD [96,102]. For example, Kaynak and Filibeli [102] processed surplus sludge with Fenton’s reagent (0.067 g Fe^2+^/g of H_2_O_2_ and 60 g H_2_O_2_/kgTS), then anaerobically digested it in an 8.5 dm^3^ laboratory digester under mesophilic conditions (5 d retention time). The VS removal was 25.4%, i.e., 1.53 times the rate achieved in the control reactor [102]. Another study [96] pre-treated AD-bound sludge with Fenton’s reagent (0.07 Fe^2+^/g of H_2_O_2_, 50 mg H_2_O_2_/kgTS at pH = 3 ), achieving 26.6% VS reduction [96].

#### 3.2.4. pH and FOS/TAC

**Figure 10:**
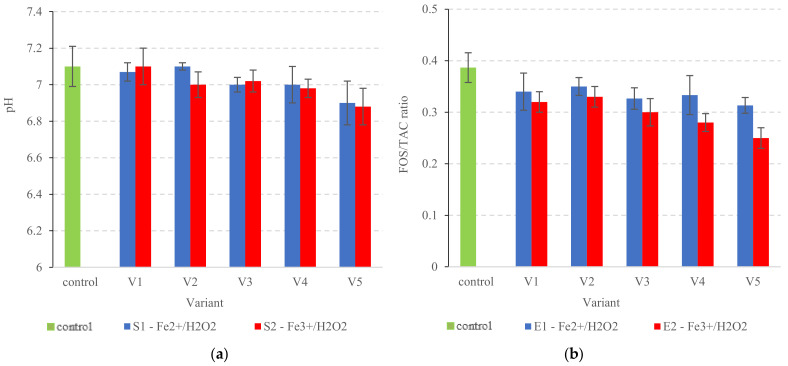
(**a**) The pH, (**b**) FOS/TAC of the digested PSS after pre-treatment.

PSS pre-treatment with Fenton’s reagent was found to have no significant effect in S1 (*p* > 0.05), with pH falling within the narrow range of 6.90 ± 0.12 to 7.10 ± 0.02 (Figure 10a). Lower pH values in the model digesters were noted for S2. In S2V5, the Fenton-like reagent triggered a pH reduction to a level of 6.88 ± 0.10 (Figure 10a). On the other hand, lower doses of Fe^3+^/H_2_O_2_ did not reduce pH significantly (*p* > 0.05), with the pH ranging from 7.1 ± 0.10 to 6.98 ± 0.05 (Figure 10a). For comparison, the digester pH was 7.10 ± 0.11 in the control group (Figure 10a). This finding is supported by Zawieja and Brzeska [95], who did not observe any significant reductions in pH during AD of Fenton-oxidized surplus sludge, noting that the sludge pH was 7.74 [95]. However, according to some authors [103], the Fenton reaction converts organic material into organic acids and can lower pH. As such, pH drops during the Fenton reactions should be controlled to optimize treatment efficiency [103]. The FOS/TAC during the digestion of non-pretreated PSS was 0.39 ± 0.03 (Figure 10b). This variable was significantly reduced by AOP, regardless of whether radical formation was mediated by Fe^2+^ or Fe^3+^ (*p* < 0.05). The change was more pronounced in S2, with FOS/TAC ranging from 0.33 ± 0.02 (S2V2) to 0.30 ± 0.03 (S2V3) (Figure 10b). Substantial reductions in FOS/TAC were observed for S2V4 and S2V5 (to 0.28 ± 0.01 and 0.25 ± 0.02, respectively) (Figure 10b). In the conventional Fenton group (Fe^2+^/H_2_O_2_), the FOS/TAC ratio remained close to the optimal level for AD across the entire range of reagent doses tested. The lowest value was recorded for S1V5 at 0.31 ± 0.01, the highest—for S1V2 at 0.35 ± 0.02 (Figure 10b). The FOS/TAC is the ratio of volatile organic acid to alkaline buffer capacity, often used to assess process stability in anaerobic digesters. Literature reports [104,105] state that FOS/TAC needs to be between 0.2 and 0.6 for stable AD, as was the case in the present study. The FOS/TAC ratio exceeding 0.6 indicates suboptimal running parameters for anaerobic microbes and reduced biogas production [104,105].

#### 3.2.5. Empirical Model and Correlations

Empirical equations were elaborated using multiple regression to estimate the methane yields. Methane production was found to correlate significantly with factors such as the dose of Fenton’s reagent, toxicity, and VS after pre-treatment. The methane production model for series 1 (4) had an estimation error of ±0.4933 and accounted for approx. 99.88% of the biogas yield variation (R^2^ coefficient of determination = 0.9988). The methane estimation model for series 2 (5) accounted for approx. 99.58% of the methane yield variation (R^2^ coefficient of determination = 0.9958) at an estimation error of ±1.3523. The level of mapping the final methane production in the developed models concerning the results obtained in the experimental work was very high, which indicated that the adopted assumptions and the practical value of the optimization procedure used were correct.

(4) METHANE S1=−57.641Fe2+H2O2−10.231 T−8.995 VS+1593.34

(5) METHANE S2=24.3628 Fe3+H2O2−4.1331 T−1.9553+698.3072

$$\mathrm{METHANE}\;–\;\mathrm{methane}\;\mathrm{production}\;({\mathrm{cm}}^{3}/\mathrm{gVS})$$

Fe2+H2O2—dose of the Fe2+/H2O2 Fenton’s reagent (g/dm3)

Fe3+H2O2—dose of the Fe3+/H2O2 Fenton’s reagent (g/dm3)

T—toxicity(%)

VS—volatile solids after processing (%TS).

**Figure 11:**
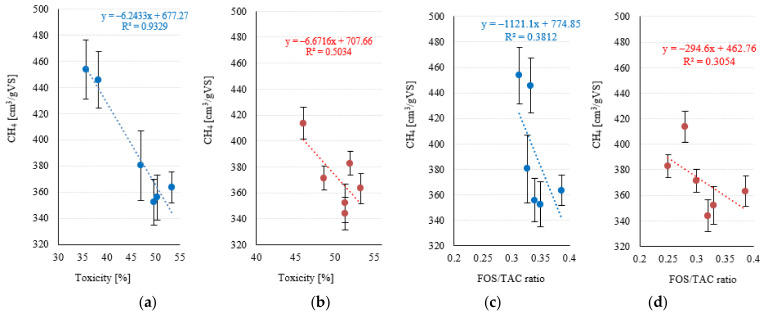
Correlations between toxicity and methane levels (**a**) series 1, (**b**) series 2; FOS/TAC ratio and methane levels (**c**) series 1, (**d**) series 2.

**Figure 12:**
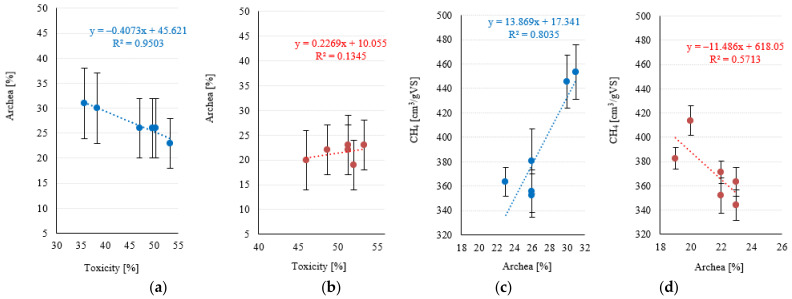
Correlations between toxicity and the share of Archaea (**a**) series 1, (**b**) series 2; the share of Archaea and methane levels (**c**) series 1, (**d**) series 2.

**Figure 13:**
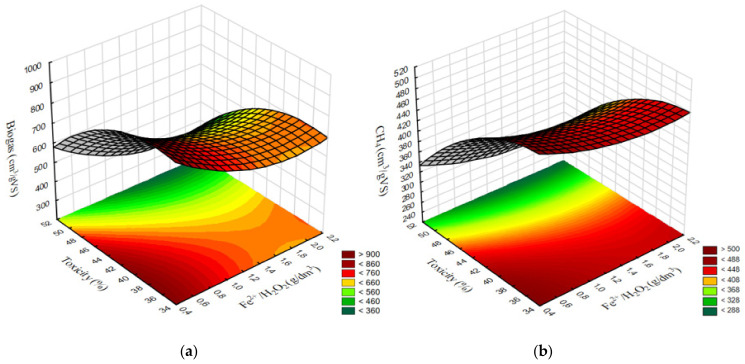
Predicted surface correlation between Fenton’s reagent dosage + toxicity and production of (**a**) biogas, (**b**) methane in series 1.

**Figure 14:**
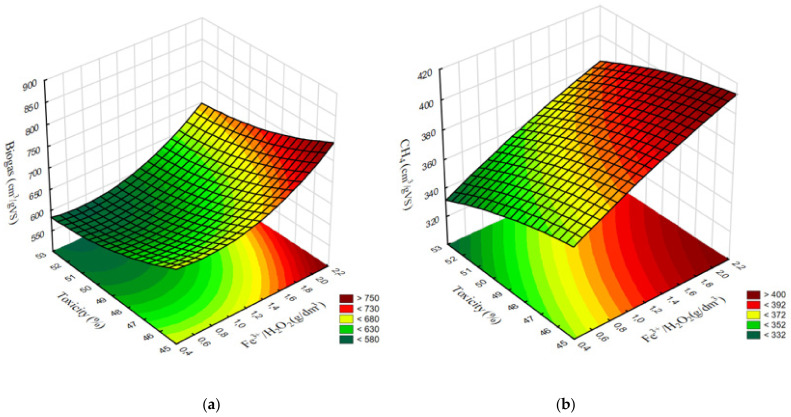
Predicted surface correlation between Fenton’s reagent dosage + toxicity and production of (**a**) biogas and (**b**) methane in series 2.

Most of the strong correlations found in series 1 pertained to the conventional Fenton reaction (Fe^2+^/H_2_O_2_). No strong and significant relationships were found for the Fe^3+^/H_2_O_2_ reagent used in series 2 (*p* > 0.05). A very strong negative association (R^2^ = 0.9329) was noted between toxicity and methane levels in series 1 (Figure 11a). In contrast, this correlation was only moderate in series 1 (R^2^ = 0.5034) (Figure 11b). Weak negative correlations (R^2^ < 0.4) were found between FOS/TAC and methane levels in both experimental series (Figure 11c,d). A very strong negative correlation (R^2^ = 0.9503) was noted between toxicity and the share of Archaea in series 1 (Figure 12a), whereas no such correlation was observed in series 2 (R^2^ = 0.1345) (Figure 12b). The only strong positive correlation (R^2^ = 0.8035) in series 1 was found between the share of Archaea and methane levels (Figure 12c), whereas the corresponding association for series 2 was only moderate (R^2^ = 0.5713) (Figure 12d). The predicted correlated effect of Fenton’s reagent dosage and toxicity on biogas and methane production is presented, respectively, in Figure 13a,b for series 1 and Figure 14a,b for series 2.

In the Fenton reagent (S1) variants, strong linear correlations between the dose of Fe^2+^/H_2_O_2_ and toxicity, presence of Archaea and CH_4_ production were revealed. In subsequent variants, a tendency to significantly reduce the toxicity of PSS and an increase in the share of Archaea in the anaerobic bacterial community was observed. These phenomena were closely correlated with the increase in the amount of biogas produced and the percentage of CH_4_ content. Unlike S1, when the like-Fenton reagent (Fe^3+^/H_2_O_2_) was used (S2), the linear relationships between the analyzed variables were disturbed. In S2, the highest chemical doses resulted in an increase in the residual H_2_O_2_ concentration in PSS, which probably resulted in an increase in toxicity. This directly affected the decrease in the average number of Archaea in the population of anaerobic bacteria, as well as the decrease in the content of CH_4_ in biogas. However, the high efficiency of biogas production was maintained. The increase in biogas synthesis efficiency in S1 (V4–V5) despite the increase in toxicity may be explained by the disintegration and destruction of the organic substrate structure as a result of AOP [106]. This could lead to an increase in the susceptibility of PSS biomass to biodegradation under anaerobic conditions [107,108]. Certainly, the process did not cause complete destruction of cellular structures, as the concentration of TOC in the dissolved phase did not reflect this. Nevertheless, the highest doses of chemical reagents tested were able to disrupt cell walls and accelerate biogas production. The positive effect of AOP on anaerobic digestion and biogas production has been proven in the literature [100,109,110]. In variants V1–V3, the toxicity was limited, which directly affected the increase in the share of Archaea in the bacterial community and the increase in the content of CH_4_ in biogas. Apparently, however, the oxidizing (disintegrating) power was too low in these pre-treatment variants to increase biogas production.

These opposite phenomena (increase in biogas production, decrease in CH_4_ content) disturbed the linear correlations between the analyzed variables in S2, which were weaker compared to the Fe^2+^/H_2_O_2_ system. The lack of strong linear relationships when the like-Fenton reagent in S2 was used makes it a method in which it is difficult to estimate the achievable final effects. This is another, apart from higher technological efficiency, argument indicating the practical advantage of AOP based on the Fe^2+^/H_2_O_2_ reagent system.

## 4. Conclusions

AOP pre-treatment of PSS using the conventional Fenton’s reagent (Fe^2+^/H_2_O_2_) and a Fenton-like reagent (Fe^3+^/H_2_O_2_) was successful in reducing dissolved organics by single-digit percentages and, most importantly—in detoxifying this substrate. Considerably better toxicity removal performance was noted for the Fe^2+^/H_2_O_2_ system. The poorer performance of the Fe^3+^/H_2_O_2_ in this regard probably stemmed from the lower oxidation capacity of the Fenton-like reaction and the relatively high levels of H_2_O_2_ residues in the PSS at the highest chemical reagent doses. The tested AOPs did not reduce the VS levels in the PSS biomass to a significant degree—a promising finding in terms of its use as an AD substrate.

Sludge pre-treated with Fe^2+^/H_2_O_2_ proved to be a better feedstock for anaerobic digestion, both with regard to biogas/methane yields and the PSS portion digested. Significantly higher values were observed for the two highest reagent doses. This was 445.7 ± 21.6 cm^3^ CH_4_/g VS (1.5 g Fe^2+^/dm^3^ and 6.0 g H_2_O_2_/dm^3^) and 453.6 ± 22.4 cm^3^ CH_4_/g VS (2.0 g Fe^2+^/dm^3^ and 8.0 g H_2_O_2_/dm^3^). The differences between these variants were not statistically significant. Therefore, due to the economical use of chemical reagents, the optimal tested dose was 1.5 g Fe^2+^/6.0 g H_2_O_2_. The use of a Fenton-like reagent (Fe^3+^/H_2_O_2_) resulted in lower AD efficiency (max. 393.7 ± 12.1 cm^3^ CH_4_/g VS), and no strong linear relationships between the analyzed variables were found. It is, therefore, a more difficult method to estimate the final effects. Research has proven that AOP can be used to improve the efficiency of AD of PSS.

Strong correlations between the tested parameters were found for the conventional Fenton reaction (Fe^2+^/H_2_O_2_). There was also a very strong negative correlation between toxicity and methane production, as well as between PSS toxicity and the share of Archaea in the microbial structure. No strong and significant correlations pertaining to reagent dosage were found for the Fe^3+^/H_2_O_2_ system.

Empirical equations were elaborated using multiple regression to estimate biogas and methane yields. Biogas and methane production was found to correlate significantly with such factors as the dose of Fenton’s reagent, toxicity, and initial VS after PSS processing.

## Figures and Tables

**Table 1:** Experimental design.

Phase 1 (P1)—AOP		
Variant (V)	Series 1 (S1)—Fe^2+^/H_2_O_2_	Series 2 (S2)—Fe^3+^/H_2_O_2_
	g/dm^3^	
V1	0.50/2.0	0.50/2.0
V2	0.75/3.0	0.75/3.0
V3	1.00/4.0	1.00/4.0
V4	1.50/6.0	1.50/6.0
V5	2.00/8.0	2.00/8.0
**Phase 2 (P2)**— each variant of AOP of PSS was tested with regard to AD performance by means of respirometric measurements in semi-batch reactors. P2 was divided into the same series (S1, S2) and variants (V1–V5) as P1.		

**Table 2:** Characteristics of the PSS and AS used in the study.

Indicator	Unit	PSS	AS
pH	-	7.3 ± 0.2	7.1 ± 0.1
Total solids (TS)	%	6.4 ± 0.4	2.2 ± 0.4
Volatile solids (VS)	%TS	78.7 ± 0.9	69.9 ± 0.6
Mineral solids (MS)	%TS	27.3 ± 0.7	30.1 ± 0.7
Total carbon (TC)	mg/gTS	430 ± 13	277 ± 11
Total organic carbon (TOC)	mg/gTS	326 ± 10	201 ± 12
Total nitrogen (TN)	mg/gTS	24 ± 3	20 ± 1.7
Total phosphorus (TP)	mg/gTS	2.1 ± 0.7	1.7 ± 0.2
Organic carbon to nitrogen ratio (C/N)	-	13.5 ± 1.4	10 ± 1.1
Protein	%TS	15.4 ± 1.9	12.2 ± 1.2
Lipids	%TS	10.1 ± 1.1	4.1 ± 0.4
Sugars	%TS	13.7 ± 2.3	1.6 ± 0.3

**Table 3:** Changes in concentrations of organic compounds (VS) and dissolved TOC in the PSS after pre-treatment.

Factor	Unit	Series (S)–Variant (V)					
		Control	S1V1	S1V2	S1V3	S1V4	S1V5
VS	Concentration (%TS)	78.7 ± 1.0	77.1 ± 2.7	76.7 ± 2.6	75.0 ± 1.1	74.4 ± 2.0	73.3 ± 2.2
	Removal (%)	-	2.0 ± 0.07	2.5 ± 0.07	4.7 ± 0.03	5.4 ± 0.08	6.8 ± 0.1
TOC	Concentration (mg/dm^3^)	326.3 ± 10.1	324.0 ± 14.7	314.0 ± 15.1	314.7 ± 9.3	302.3 ± 20.2	296.1 ± 5.0
	Removal (%)	-	0.7 ± 0.03	3.8 ± 0.04	3.6 ± 0.02	7.3 ± 0.05	9.2 ± 0.02
**Factor**	**Unit**	**Series (S)–Variant (V)**					
		**Control**	**S2V1**	**S2V2**	**S2V3**	**S2V4**	**S2V5**
VS	Concentration (%TS)	78.7 ± 1.0	78.5 ± 0.4	78.4 ± 1.2	77.0 ± 0.3	77.2 ± 1.1	76.3 ± 0.6
	Removal (%)	-	0.2 ± 0.02	0.3 ± 0.03	2.1 ± 0.01	1.9 ± 0.04	2.9 ± 0.02
TOC	Concentration (mg/dm^3^)	326.3 ± 10.1	325.7 ± 8.1	318.0 ± 6.6	321.7 ± 12.8	305.0 ± 10.6	303.0 ± 8.5
	Removal (%)	-	0.2 ± 0.01	2.5 ± 0.02	1.4 ± 0.03	6.5 ± 0.03	7.1 ± 0.02

**Table 4:** Portion digested and biogas/CH_4_ production in relation to VS load removed.

Factor	Unit	Series (S)–Variant (V)					
		Control	S1V1	S1V2	S1V3	S1V4	S1V5
$\eta_{F}$	%	43.15 ± 2.2	43.18 ± 4.1	35.17 ± 4.2	47.69 ± 3.8	59.18 ± 2.3	58.50 ± 3.2
$Y_{b}^{VS_{rem.}}$	cm^3^/g*VS_rem._*	1455 ± 10	1480 ± 13	1708 ± 15	1309 ± 11	1184 ± 15	1182 ± 18
$Y_{CH_{4}}^{VS_{rem.}}$	cm^3^/g*VS_rem._*	869 ± 8	854 ± 5	1047 ± 9	816 ± 8	762 ± 7	780 ± 9
**Factor**	**Unit**	**Series (S)–Variant (V)**					
		**Control**	**S2V1**	**S2V2**	**S2V3**	**S2V4**	**S2V5**
$\eta_{F}$	%	43.15 ± 2.2	38.21 ± 3.1	40.21 ± 3.0	48.05 ± 2.2	49.04 ± 3.4	45.58 ± 2.1
$Y_{b}^{VS_{rem.}}$	cm^3^/g*VS_rem._*	1455 ± 10	1594 ± 18	1497 ± 13	1309 ± 17	1431 ± 15	1509 ± 19
$Y_{CH_{4}}^{VS_{rem.}}$	cm^3^/g*VS_rem._*	869 ± 8	935 ± 9	903 ± 5	794 ± 7	825 ± 8	860 ± 8

**Table 5:** Microbial populations in AS across experimental variants.

Taxonomic Group	S1–Fe^2+^/H_2_O_2_					
	Control	V1	V2	V3	V4	V6
Bacteria (EUB338)	69 ± 10	70 ± 11	71 ± 12	69 ± 10	68 ± 13	69 ± 11
Archaea (ARC915)	23 ± 5	26 ± 6	26 ± 6	26 ± 6	30 ± 7	31 ± 7
*Methanosarcinaceae* (MSMX860)	11 ± 4	11 ± 4	13 ± 6	12 ± 5	13 ± 5	14 ± 6
*Methanosaeta* (MX825)	6 ± 2	7 ± 3	8 ± 3	8 ± 4	10 ± 4	10 ± 4
**Taxonomic Group**	**S2–Fe^3+^/H_2_O_2_**					
	**Control**	**V1**	**V2**	**V3**	**V4**	**V6**
Bacteria (EUB338)	69 ± 10	68 ± 10	70 ± 14	69 ± 12	67 ± 11	68 ± 12
Archaea (ARC915)	23 ± 5	23 ± 6	22 ± 5	22 ± 5	20 ± 6	19 ± 5
*Methanosarcinaceae* (MSMX860)	11 ± 5	12 ± 6	10 ± 4	11 ± 5	9 ± 4	9 ± 4
*Methanosaeta* (MX825)	6 ± 2	8 ± 3	9 ± 4	8 ± 3	6 ± 2	7 ± 3
